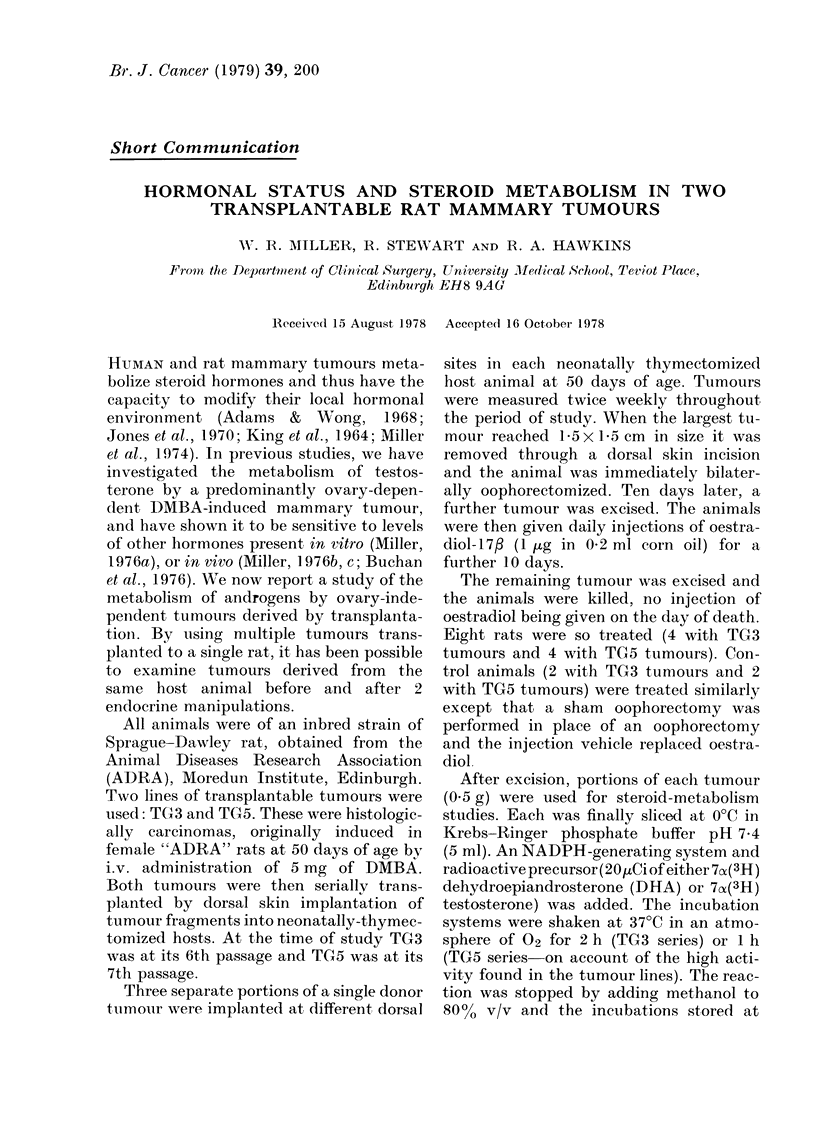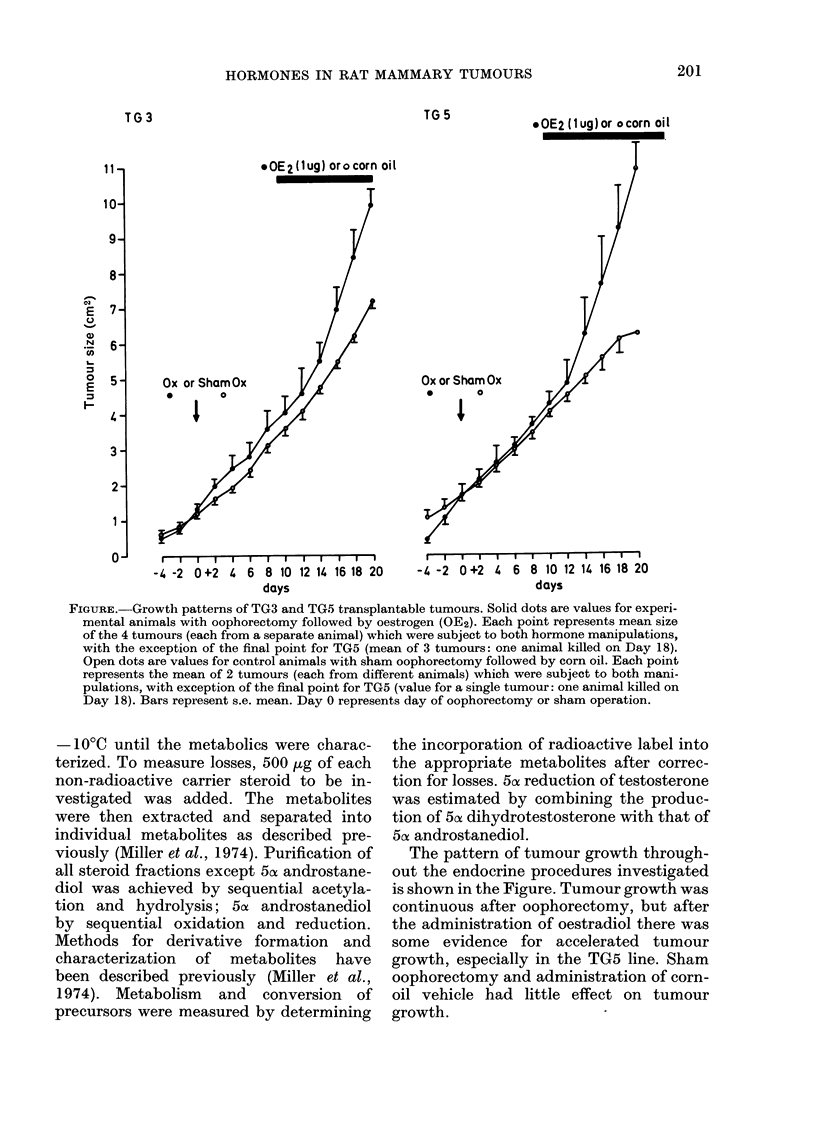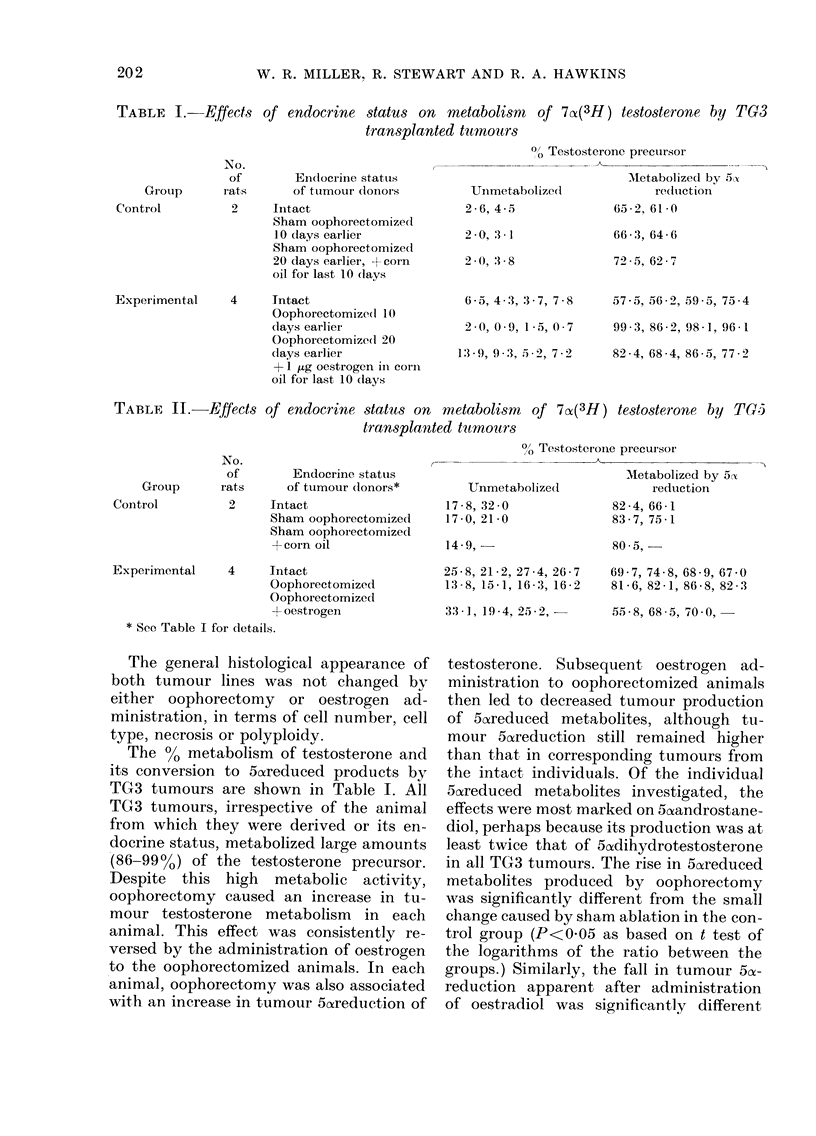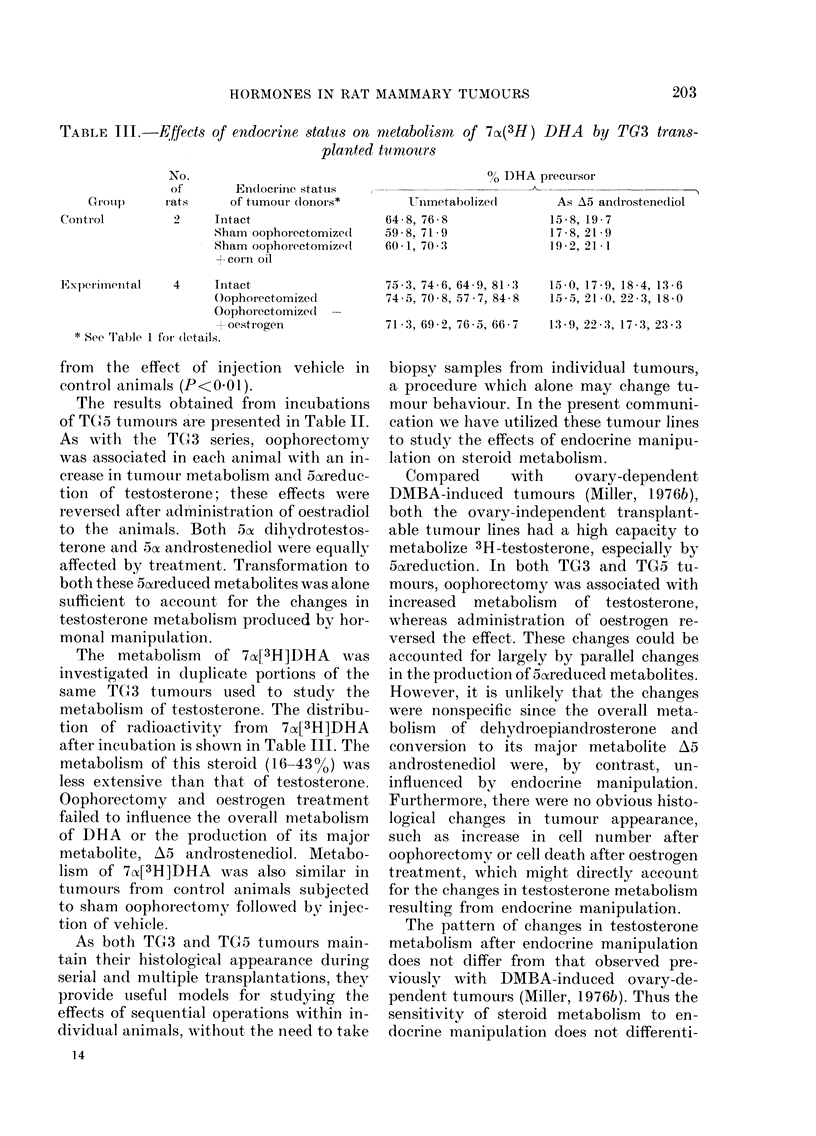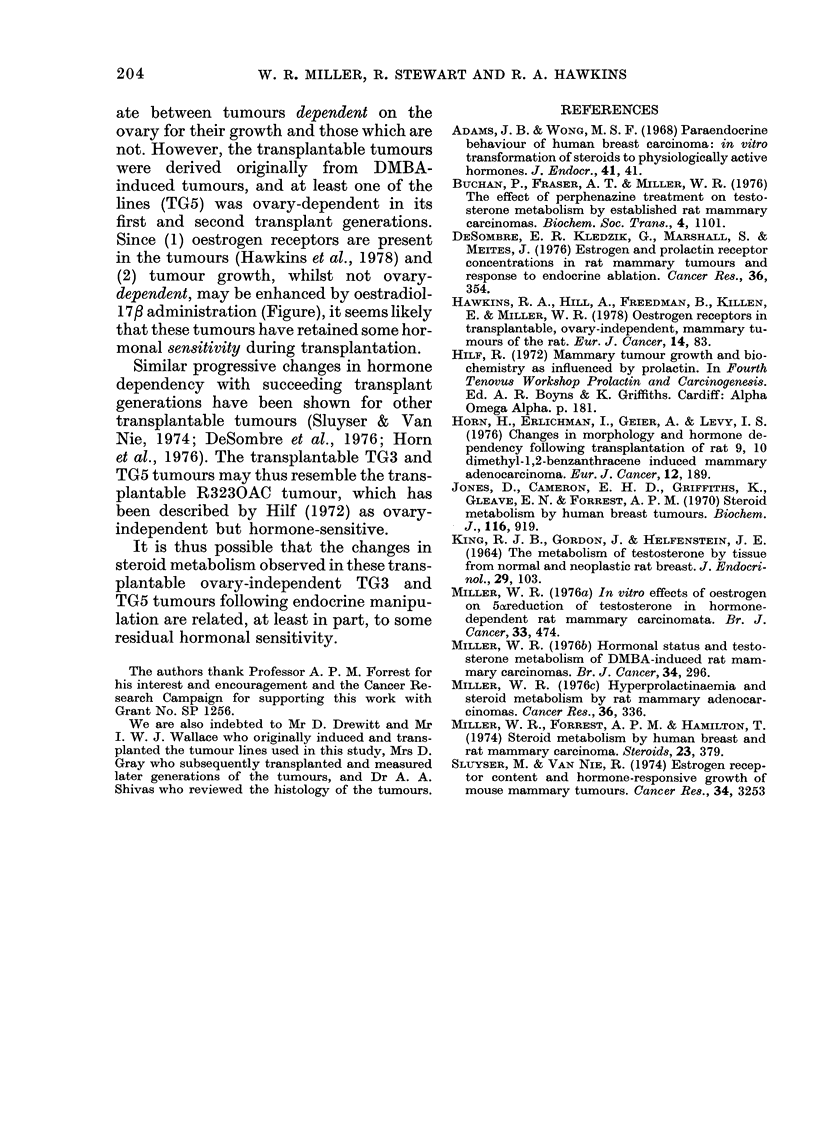# Hormonal status and steroid metabolism in two transplantable rat mammary tumours.

**DOI:** 10.1038/bjc.1979.32

**Published:** 1979-02

**Authors:** W. R. Miller, R. Stewart, R. A. Hawkins


*Br. J. Cancer* (1979) **39**, 200

***Short Communication***

### HORMONAL STATUS AND STEROID METABOLISM IN TWO TRANSPLANTABLE RAT MAMMARY TUMOURS

W. R. MILLER, R. STEWART AND R. A. HAWKINS

*From the Department of Clinical Surgery, University Medical School, Teviot Place, Edinburgh EH8 9AG*

Received 15 August 1978 Accepted 16 October 1978

HUMAN and rat mammary tumours metabolize steroid hormones and thus have the capacity to modify their local hormonal environment (Adams & Wong, 1968; Jones *et al.*, 1970; King *et al.*, 1964; Miller *et al.*, 1974). In previous studies, we have investigated the metabolism of testosterone by a predominantly ovary-dependent DMBA-induced mammary tumour, and have shown it to be sensitive to levels of other hormones present *in vitro* (Miller, 1976*a*), or *in vivo* (Miller, 1976*b*, *c*; Buchan *et al.*, 1976). We now report a study of the metabolism of androgens by ovary-independent tumours derived by transplantation. By using multiple tumours transplanted to a single rat, it has been possible to examine tumours derived from the same host animal before and after 2 endocrine manipulations.

All animals were of an inbred strain of Sprague–Dawley rat, obtained from the Animal Diseases Research Association (ADRA), Moredun Institute, Edinburgh. Two lines of transplantable tumours were used: TG3 and TG5. These were histologically carcinomas, originally induced in female "ADRA" rats at 50 days of age by i.v. administration of 5 mg of DMBA. Both tumours were then serially transplanted by dorsal skin implantation of tumour fragments into neonatally-thymectomized hosts. At the time of study TG3 was at its 6th passage and TG5 was at its 7th passage.

Three separate portions of a single donor tumour were implanted at different dorsal sites in each neonatally thymectomized host animal at 50 days of age. Tumours were measured twice weekly throughout the period of study. When the largest tumour reached $1 \cdot 5 \times 1 \cdot 5$ cm in size it was removed through a dorsal skin incision and the animal was immediately bilaterally oophorectomized. Ten days later, a further tumour was excised. The animals were then given daily injections of oestradiol-17$\beta$ (1 $\mu$g in 0·2 ml corn oil) for a further 10 days.

The remaining tumour was excised and the animals were killed, no injection of oestradiol being given on the day of death. Eight rats were so treated (4 with TG3 tumours and 4 with TG5 tumours). Control animals (2 with TG3 tumours and 2 with TG5 tumours) were treated similarly except that a sham oophorectomy was performed in place of an oophorectomy and the injection vehicle replaced oestradiol.

After excision, portions of each tumour (0·5 g) were used for steroid-metabolism studies. Each was finally sliced at 0°C in Krebs–Ringer phosphate buffer pH 7·4 (5 ml). An NADPH-generating system and radioactive precursor (20 $\mu$Ci of either 7$\alpha$($^3$H) dehydroepiandrosterone (DHA) or 7$\alpha$($^3$H) testosterone) was added. The incubation systems were shaken at 37°C in an atmosphere of $O_2$ for 2 h (TG3 series) or 1 h (TG5 series—on account of the high activity found in the tumour lines). The reaction was stopped by adding methanol to 80% v/v and the incubations stored at

FIGURE.—Growth patterns of TG3 and TG5 transplantable tumours. Solid dots are values for experimental animals with oophorectomy followed by oestrogen ($OE_2$). Each point represents mean size of the 4 tumours (each from a separate animal) which were subject to both hormone manipulations, with the exception of the final point for TG5 (mean of 3 tumours: one animal killed on Day 18). Open dots are values for control animals with sham oophorectomy followed by corn oil. Each point represents the mean of 2 tumours (each from different animals) which were subject to both manipulations, with exception of the final point for TG5 (value for a single tumour: one animal killed on Day 18). Bars represent s.e. mean. Day 0 represents day of oophorectomy or sham operation.

—10°C until the metabolics were characterized. To measure losses, 500 $\mu$g of each non-radioactive carrier steroid to be investigated was added. The metabolites were then extracted and separated into individual metabolites as described previously (Miller *et al.*, 1974). Purification of all steroid fractions except 5α androstanediol was achieved by sequential acetylation and hydrolysis; 5α androstanediol by sequential oxidation and reduction. Methods for derivative formation and characterization of metabolites have been described previously (Miller *et al.*, 1974). Metabolism and conversion of precursors were measured by determining the incorporation of radioactive label into the appropriate metabolites after correction for losses. 5α reduction of testosterone was estimated by combining the production of 5α dihydrotestosterone with that of 5α androstanediol.

The pattern of tumour growth throughout the endocrine procedures investigated is shown in the Figure. Tumour growth was continuous after oophorectomy, but after the administration of oestradiol there was some evidence for accelerated tumour growth, especially in the TG5 line. Sham oophorectomy and administration of corn-oil vehicle had little effect on tumour growth.

TABLE I.—*Effects of endocrine status on metabolism of 7α($^3H$) testosterone by TG3 transplanted tumours*

| Group | No. of rats | Endocrine status of tumour donors | % Testosterone precursor: Unmetabolized | % Testosterone precursor: Metabolized by 5α reduction |
|---|---|---|---|---|
| Control | 2 | Intact | 2·6, 4·5 | 65·2, 61·0 |
| | | Sham oophorectomized 10 days earlier | 2·0, 3·1 | 66·3, 64·6 |
| | | Sham oophorectomized 20 days earlier, +corn oil for last 10 days | 2·0, 3·8 | 72·5, 62·7 |
| Experimental | 4 | Intact | 6·5, 4·3, 3·7, 7·8 | 57·5, 56·2, 59·5, 75·4 |
| | | Oophorectomized 10 days earlier | 2·0, 0·9, 1·5, 0·7 | 99·3, 86·2, 98·1, 96·1 |
| | | Oophorectomized 20 days earlier +1 μg oestrogen in corn oil for last 10 days | 13·9, 9·3, 5·2, 7·2 | 82·4, 68·4, 86·5, 77·2 |

TABLE II.—*Effects of endocrine status on metabolism of 7α($^3H$) testosterone by TG5 transplanted tumours*

| Group | No. of rats | Endocrine status of tumour donors* | % Testosterone precursor: Unmetabolized | % Testosterone precursor: Metabolized by 5α reduction |
|---|---|---|---|---|
| Control | 2 | Intact | 17·8, 32·0 | 82·4, 66·1 |
| | | Sham oophorectomized | 17·0, 21·0 | 83·7, 75·1 |
| | | Sham oophorectomized +corn oil | 14·9, — | 80·5, — |
| Experimental | 4 | Intact | 25·8, 21·2, 27·4, 26·7 | 69·7, 74·8, 68·9, 67·0 |
| | | Oophorectomized | 13·8, 15·1, 16·3, 16·2 | 81·6, 82·1, 86·8, 82·3 |
| | | Oophorectomized +oestrogen | 33·1, 19·4, 25·2, — | 55·8, 68·5, 70·0, — |

* See Table I for details.

The general histological appearance of both tumour lines was not changed by either oophorectomy or oestrogen administration, in terms of cell number, cell type, necrosis or polyploidy.

The % metabolism of testosterone and its conversion to 5αreduced products by TG3 tumours are shown in Table I. All TG3 tumours, irrespective of the animal from which they were derived or its endocrine status, metabolized large amounts (86–99%) of the testosterone precursor. Despite this high metabolic activity, oophorectomy caused an increase in tumour testosterone metabolism in each animal. This effect was consistently reversed by the administration of oestrogen to the oophorectomized animals. In each animal, oophorectomy was also associated with an increase in tumour 5αreduction of testosterone. Subsequent oestrogen administration to oophorectomized animals then led to decreased tumour production of 5αreduced metabolites, although tumour 5αreduction still remained higher than that in corresponding tumours from the intact individuals. Of the individual 5αreduced metabolites investigated, the effects were most marked on 5αandrostanediol, perhaps because its production was at least twice that of 5αdihydrotestosterone in all TG3 tumours. The rise in 5αreduced metabolites produced by oophorectomy was significantly different from the small change caused by sham ablation in the control group ($P<0{\cdot}05$ as based on $t$ test of the logarithms of the ratio between the groups.) Similarly, the fall in tumour 5α-reduction apparent after administration of oestradiol was significantly different

TABLE III.—*Effects of endocrine status on metabolism of 7α($^3H$) DHA by TG3 transplanted tumours*

| Group | No. of rats | Endocrine status of tumour donors* | % DHA precursor: Unmetabolized | % DHA precursor: As Δ5 androstenediol |
|---|---|---|---|---|
| Control | 2 | Intact | 64·8, 76·8 | 15·8, 19·7 |
| | | Sham oophorectomized | 59·8, 71·9 | 17·8, 21·9 |
| | | Sham oophorectomized +corn oil | 60·1, 70·3 | 19·2, 21·1 |
| Experimental | 4 | Intact | 75·3, 74·6, 64·9, 81·3 | 15·0, 17·9, 18·4, 13·6 |
| | | Oophorectomized | 74·5, 70·8, 57·7, 84·8 | 15·5, 21·0, 22·3, 18·0 |
| | | Oophorectomized — +oestrogen | 71·3, 69·2, 76·5, 66·7 | 13·9, 22·3, 17·3, 23·3 |

* See Table 1 for details.

from the effect of injection vehicle in control animals ($P<0·01$).

The results obtained from incubations of TG5 tumours are presented in Table II. As with the TG3 series, oophorectomy was associated in each animal with an increase in tumour metabolism and 5αreduction of testosterone; these effects were reversed after administration of oestradiol to the animals. Both 5α dihydrotestosterone and 5α androstenediol were equally affected by treatment. Transformation to both these 5αreduced metabolites was alone sufficient to account for the changes in testosterone metabolism produced by hormonal manipulation.

The metabolism of 7α[$^3$H]DHA was investigated in duplicate portions of the same TG3 tumours used to study the metabolism of testosterone. The distribution of radioactivity from 7α[$^3$H]DHA after incubation is shown in Table III. The metabolism of this steroid (16–43%) was less extensive than that of testosterone. Oophorectomy and oestrogen treatment failed to influence the overall metabolism of DHA or the production of its major metabolite, Δ5 androstenediol. Metabolism of 7α[$^3$H]DHA was also similar in tumours from control animals subjected to sham oophorectomy followed by injection of vehicle.

As both TG3 and TG5 tumours maintain their histological appearance during serial and multiple transplantations, they provide useful models for studying the effects of sequential operations within individual animals, without the need to take biopsy samples from individual tumours, a procedure which alone may change tumour behaviour. In the present communication we have utilized these tumour lines to study the effects of endocrine manipulation on steroid metabolism.

Compared with ovary-dependent DMBA-induced tumours (Miller, 1976*b*), both the ovary-independent transplantable tumour lines had a high capacity to metabolize $^3$H-testosterone, especially by 5αreduction. In both TG3 and TG5 tumours, oophorectomy was associated with increased metabolism of testosterone, whereas administration of oestrogen reversed the effect. These changes could be accounted for largely by parallel changes in the production of 5αreduced metabolites. However, it is unlikely that the changes were nonspecific since the overall metabolism of dehydroepiandrosterone and conversion to its major metabolite Δ5 androstenediol were, by contrast, uninfluenced by endocrine manipulation. Furthermore, there were no obvious histological changes in tumour appearance, such as increase in cell number after oophorectomy or cell death after oestrogen treatment, which might directly account for the changes in testosterone metabolism resulting from endocrine manipulation.

The pattern of changes in testosterone metabolism after endocrine manipulation does not differ from that observed previously with DMBA-induced ovary-dependent tumours (Miller, 1976*b*). Thus the sensitivity of steroid metabolism to endocrine manipulation does not differentiate between tumours *dependent* on the ovary for their growth and those which are not. However, the transplantable tumours were derived originally from DMBA-induced tumours, and at least one of the lines (TG5) was ovary-dependent in its first and second transplant generations. Since (1) oestrogen receptors are present in the tumours (Hawkins *et al.*, 1978) and (2) tumour growth, whilst not ovary-*dependent*, may be enhanced by oestradiol-17$\beta$ administration (Figure), it seems likely that these tumours have retained some hormonal *sensitivity* during transplantation.

Similar progressive changes in hormone dependency with succeeding transplant generations have been shown for other transplantable tumours (Sluyser & Van Nie, 1974; DeSombre *et al.*, 1976; Horn *et al.*, 1976). The transplantable TG3 and TG5 tumours may thus resemble the transplantable R3230AC tumour, which has been described by Hilf (1972) as ovary-independent but hormone-sensitive.

It is thus possible that the changes in steroid metabolism observed in these transplantable ovary-independent TG3 and TG5 tumours following endocrine manipulation are related, at least in part, to some residual hormonal sensitivity.

The authors thank Professor A. P. M. Forrest for his interest and encouragement and the Cancer Research Campaign for supporting this work with Grant No. SP 1256.

We are also indebted to Mr D. Drewitt and Mr I. W. J. Wallace who originally induced and transplanted the tumour lines used in this study, Mrs D. Gray who subsequently transplanted and measured later generations of the tumours, and Dr A. A. Shivas who reviewed the histology of the tumours.

### REFERENCES

Adams, J. B. & Wong, M. S. F. (1968) Paraendocrine behaviour of human breast carcinoma: *in vitro* transformation of steroids to physiologically active hormones. *J. Endocr.*, **41**, 41.

Buchan, P., Fraser, A. T. & Miller, W. R. (1976) The effect of perphenazine treatment on testosterone metabolism by established rat mammary carcinomas. *Biochem. Soc. Trans.*, **4**, 1101.

DeSombre, E. R. Kledzik, G., Marshall, S. & Meites, J. (1976) Estrogen and prolactin receptor concentrations in rat mammary tumours and response to endocrine ablation. *Cancer Res.*, **36**, 354.

Hawkins, R. A., Hill, A., Freedman, B., Killen, E. & Miller, W. R. (1978) Oestrogen receptors in transplantable, ovary-independent, mammary tumours of the rat. *Eur. J. Cancer*, **14**, 83.

Hilf, R. (1972) Mammary tumour growth and biochemistry as influenced by prolactin. In *Fourth Tenovus Workshop Prolactin and Carcinogenesis.* Ed. A. R. Boyns & K. Griffiths. Cardiff: Alpha Omega Alpha. p. 181.

Horn, H., Erlichman, I., Geier, A. & Levy, I. S. (1976) Changes in morphology and hormone dependency following transplantation of rat 9, 10 dimethyl-1,2-benzanthracene induced mammary adenocarcinoma. *Eur. J. Cancer*, **12**, 189.

Jones, D., Cameron, E. H. D., Griffiths, K., Gleave, E. N. & Forrest, A. P. M. (1970) Steroid metabolism by human breast tumours. *Biochem. J.*, **116**, 919.

King, R. J. B., Gordon, J. & Helfenstein, J. E. (1964) The metabolism of testosterone by tissue from normal and neoplastic rat breast. *J. Endocrinol.*, **29**, 103.

Miller, W. R. (1976*a*) *In vitro* effects of oestrogen on 5$\alpha$reduction of testosterone in hormone-dependent rat mammary carcinomata. *Br. J. Cancer*, **33**, 474.

Miller, W. R. (1976*b*) Hormonal status and testosterone metabolism of DMBA-induced rat mammary carcinomas. *Br. J. Cancer*, **34**, 296.

Miller, W. R. (1976*c*) Hyperprolactinaemia and steroid metabolism by rat mammary adenocarcinomas. *Cancer Res.*, **36**, 336.

Miller, W. R., Forrest, A. P. M. & Hamilton, T. (1974) Steroid metabolism by human breast and rat mammary carcinoma. *Steroids*, **23**, 379.

Sluyser, M. & Van Nie, R. (1974) Estrogen receptor content and hormone-responsive growth of mouse mammary tumours. *Cancer Res.*, **34**, 3253